# Effects of Shape Anisotropy on Hard–Soft Exchange-Coupled Permanent Magnets

**DOI:** 10.3390/nano12081261

**Published:** 2022-04-08

**Authors:** Zhi Yang, Yuanyuan Chen, Weiqiang Liu, Yatao Wang, Yuqing Li, Dongtao Zhang, Qingmei Lu, Qiong Wu, Hongguo Zhang, Ming Yue

**Affiliations:** Faculty of Materials and Manufacturing, Key Laboratory of Advanced Functional Materials, Ministry of Education of China, Beijing University of Technology, Beijing 100124, China; yangzhi@bjut.edu.cn (Z.Y.); chenyuanyuan415@126.com (Y.C.); ziyu0023@163.com (Y.W.); yqli@bjut.edu.cn (Y.L.); zdt@bjut.edu.cn (D.Z.); qmlu@bjut.edu.cn (Q.L.); wuqiong0506@bjut.edu.cn (Q.W.); hgzhang@bjut.edu.cn (H.Z.)

**Keywords:** permanent magnets, micromagnetic simulation, exchange-coupling, shape anisotropy, core/shell structure

## Abstract

Exchange-coupled magnets are promising candidates for a new generation of permanent magnets. Here, we investigated the effect of soft magnetic shell thickness and the aspect ratio of the hard magnetic core on the magnetic properties for isolated core/shell cylinder exchange-coupled magnets, as well as the packing effect of the cylindrical array via a micromagnetic simulation method. It was found that the shape anisotropy contributions to the magnetic properties in the cylindrical core/shell exchange-coupled magnets are closely related to the thickness of the soft magnetic shell. When the soft magnetic shell is thin, the magnetic properties are dominated by the hard–soft exchange coupling effects, and the contributions of shape anisotropy are quite limited. When the soft magnetic shell is relatively thick, utilizing shape anisotropy would be an effective method to improve the magnetic performance of hard–soft exchange-coupled magnets. The present work provides an in-depth fundamental understanding of the underlying magnetization reversal mechanism. This work could be useful for designing high-performance permanent magnets and avoiding pitfalls.

## 1. Introduction

Rare-earth permanent magnets bearing strong energy density have become vital components of modern electromagnetic technology [1,2,3], which are increasingly demanded in an impressive range of applications, including energy generation and conversion, as well as information storage and processing [4]. Today’s energy product holder is Nd_2_Fe_14_B with Dy addition, which exhibits a high coercivity even at 373 K [5]. However, due to the global trade situation, rare-earth is in short supply [6], in particular, Dy, which is expensive and has low reserves even by rare-earth standards. Over the past decades, great efforts have been devoted to developing permanent magnets with higher performance and lower costs to solve this dilemma [7,8].

Exchange-coupled magnets are promising candidates for a new generation of permanent magnets that have drawn increasing attention [9]. These magnets contain two main magnetic phases, i.e., the high-anisotropy hard magnetic phase, and the high-saturation-magnetization soft magnetic phase, which are coupled via exchange interaction on the nanometer scale. The exchange-coupled magnets offer the unique opportunity to simultaneously utilize the high coercivity of the hard magnetic phase and the high saturation magnetization of the soft magnetic phase, and then to achieve high maximum magnetic energy product (up to slightly higher than 1 MJ m^−3^, or twice the value for Nd_2_Fe_14_B) [10,11]. Meanwhile, the exchange-couple principle could also provide flexibility in material selection for the constituent hard and soft phases, which represents a possible solution for the supply risk of the strategic rare-earth elements [12]. Many studies have been conducted to improve the coercivity and magnetic energy product of the exchange-coupled magnets by controlling morphological and geometric parameters [8,13,14,15]. Nevertheless, with the increase in the soft magnetic phase, the remanence of the system increases at the expense of the coercivity [5], and there is a trade-off between the coercivity and remnant magnetization for an optimal magnetic energy product [4], which remains a major challenge for exploiting high-performance permanent magnets. 

In a single domain state at the nanometer scale, coercivity substantially higher than the magneto-crystalline anisotropy field could be achieved if the shape anisotropy can be enhanced because the shape anisotropy can provide an additional resource of coercivity by forming an effective anisotropy field [4,16,17,18]. For example, the Co nanowires with a high coercivity of ~1 T can be synthesized via numerous methods, including template-assisted electrodeposition, electroless deposition under an external magnetic field, and chemical synthesis [18]. Thus, the magnetic performance of the traditional hard–soft exchange-coupled magnets could be further improved by utilizing shape anisotropy. Furthermore, the 1-D nanostructured materials (nanowire, or nanorod) with enhanced shape anisotropy could prevent aggregation due to their unique shape characteristics, which can also be beneficial for more sufficient exchange-coupling compared to the traditional exchange-coupled nanoparticles [13]. 

Currently, micromagnetic simulations are widely used to study the magnetization phenomena on the intermediate scale between the quantum mechanical scale of individual atoms and the macroscale, such as modeling domain structures [19]. Herein, micromagnetic simulation provides a valuable tool to design exchange-coupled magnets, which is very helpful in terms of reducing the research cost and accelerating the design process [20,21]. In this work, according to the typical fabrication process of cylindrical hard core/soft shell exchange-coupled permanent magnets, we performed micromagnetic simulations. The effect of soft magnetic shell thickness and the aspect ratio of the hard magnetic core on the magnetic properties for the isolated core/shell cylinder exchange-coupled magnets was systematically investigated, and the underlying magnetization reversal mechanisms were discussed. In addition, considering that the entire magnets are assembled using amounts of isolated nanostructure units, the packing effect of the cylindrical array was also investigated. Our work may serve as the foundation for exploiting high-performance permanent magnets in the future.

## 2. Materials and Methods

**Figure 1:**
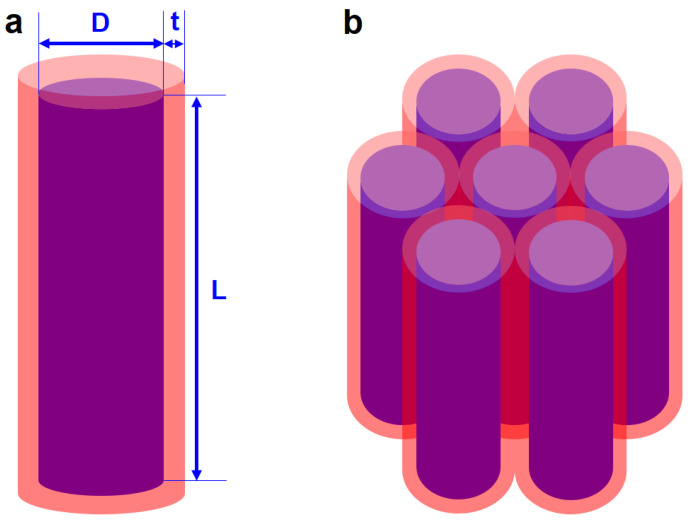
(**a**) Schematic illustration of the cylindrical core/shell structure. The outer shell represents the soft magnetic phase with thickness *t*, while the inner core represents the hard magnetic phase with diameter *D* and length *L*. (**b**) Schematic illustration of the hexagonal close-packed cylindrical array. The applied field and easy axis of the hard magnetic phase are parallel to the cylinder axis.

Micromagnetic simulations were carried out using the micromagnetic simulator OOMMF (Object Oriented MicroMagnetic Framework) [22] by dynamically solving the Landau–Lifshitz–Gillbert (LLG) equation [23,24] within the framework of the finite difference method [25]:

(1) $$\frac{dM}{dt}=-\left|\bar{\gamma }\right|M\times H_{eff}-\frac{\left|\bar{\gamma }\right|\alpha }{M_{s}}M\times (M\times H_{eff})$$

where *α* is the damping constant and $\left|\bar{\gamma }\right|$ is the gyromagnetic ratio, satisfying $\left|\bar{\gamma }\right|=\frac{\left|\gamma \right|}{1+\alpha^{2}}$. The damping constant of *α =* 0.5 has been used for the simulation of the quasi-static case [18,19,26]. Considering that the typical bottom-up assembly fabrication of cylindrical core/shell nanostructured hard–soft exchange-coupled magnets involves two stages—preparation of the precursor hard magnetic cylinder (nanowire or nanorod), and subsequent deposition of the soft magnetic shell [13]—an isolated cylindrical core/shell nanostructured micromagnetic model was used, as shown in Figure 1a. The core/shell nanostructure has a hard magnetic core surrounded by a soft magnetic shell. The diameter and length of the hard magnetic cylinder is denoted as *D* and *L*, respectively, while the thickness of the soft magnetic shell is denoted as *t*. To investigate the contributions of exchange-coupling and shape anisotropy to the magnetic properties, we calculated the demagnetization curves of the cylindrical core/shell nanostructure with various shell thickness (*t* = 1, 2, 3, 4, 5 nm) and aspect ratio (*L*/*D* = 1, 2, 3, 4, 5, 6, 7, 8, 9, 10, and *D* = 10 nm). Both the applied field *H* (from +14 T to −14 T) and the magnetocrystalline anisotropic easy axis of the hard magnetic phase are assumed to be paralleled to the longitudinal direction (*z*-axis direction) of the cylinder. The simulation mesh size of 1 nm × 1 nm × 1 nm for *t* = 3, 4, 5 nm, and 0.5 nm × 0.5 nm × 0.5 nm for *t* = 1, 2 nm were used. To examine the effect of packing [12], hexagonal close-packed cylindrical array structures were used, as illustrated in Figure 1b. For effectively exchange-coupling, it is well known that a soft magnetic layer should be thinner than twice the domain-wall width of the hard magnetic phase. The Sm_2_Co_17_ exhibits a larger domain-wall width, as compared to SmCo_5_ and Nd_2_Fe_14_B, which is more flexible for exchange coupling [13]. Here, we selected Sm_2_Co_17_ and Fe_65_Co_35_ as the hard magnetic phase and soft magnetic phase, respectively, and the magnetic parameters including anisotropy *K*_1_, saturation magnetization *M*_s_ and exchange constant *A* of Sm_2_Co_17_ [27] and Fe_65_Co_35_ [28] are as follows: *K*_1_ = 5 MJ m^−3^, *M*_s_ = 1.05 MA m^−1^, *A* = 14 pJ m^−1^ for the Sm_2_Co_17_ alloy, and *K*_1_ = 0.02 MJ m^−3^, *M*_s_ = 1.95 MA m^−1^, *A* = 16.7 pJ m^−1^. The guidelines revealed in the present work are not believed to be limited to the Sm_2_Co_17_/Fe_65_Co_35_ magnets but could be applied to other exchange-coupled magnets with different constituent hard and soft phases.

## 3. Results and Discussion

### 3.1. Effect of Soft Magnetic Shell on Magnetic Properties

**Figure 2:**
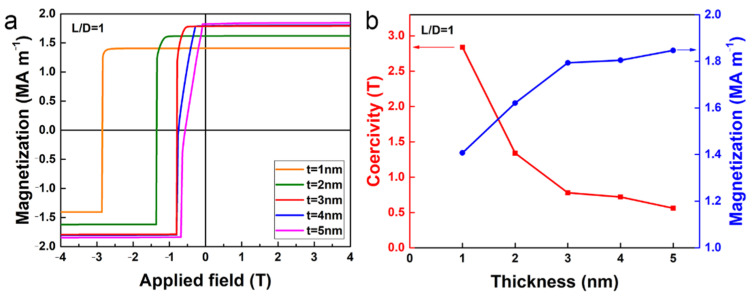
(**a**) Calculated demagnetization curves of the core/shell cylinder for different thicknesses of soft magnetic shell *t*, with aspect ratio *L*/*D* = 1. (**b**) The intrinsic coercivity (red) and saturation magnetization (blue) of the core/shell cylinder shown as a function of *t*.

To explore the effect of the soft magnetic shell thickness on magnetic properties, the demagnetization curves of the isolated core/shell cylinder with aspect ratio *L*/*D* = 1 were calculated; the results are shown in Figure 2a. It should be noted that the aspect ratio is defined as the ratio between the diameter and length of the hard magnetic core, instead of the entire core/shell cylinder. Clearly, the shape of the demagnetization curves strongly depends on the thickness of the soft magnetic shell. As the thickness of the soft magnetic shell increases, the magnetization reversal is clearly separated into two parts (i.e., the slight decrease in magnetization at the lower applied field, and the jump at the higher applied field), which deteriorates the squareness. This is because the magnetization reversal is expected to start from the soft magnetic phase prior to the magnetization of hard magnetic phase switching, as shown below. The saturation magnetization (*M*_s_) in such soft–hard exchange-coupled magnets usually follows the general mixing rule, i.e., $M_{s}=V_{{}^{\mathrm{soft}}}\times M_{s}{}^{\mathrm{soft}}+V_{{}^{\mathrm{hard}}}\times M_{s}{}^{\mathrm{hard}}$, where *M*_s_^soft^ and *M*_s_^hard^ are the saturation magnetization of the soft magnetic phase and the hard magnetic phase, respectively; *V*_soft_ and *V*_hard_ are the volume fraction of the soft magnetic phase and the hard magnetic phase, respectively. As seen from Figure 2a, the *M*_s_ increases with *t* increasing. It should be noted that saturation magnetization is an intrinsic material property, which is decisive for the remanence *M*_r_ and the maximum magnetic energy product (*BH*)_max_ [29]. The intrinsic coercivity *H*_ci_ and the saturation magnetization determined from the demagnetization curves are shown as a function of soft magnetic shell thickness *t* in Figure 2b. As seen from Figure 2b, the intrinsic coercivity *H*_ci_ decreases monotonously with *t* increasing. The exchange-coupled soft magnetic phase enhances the saturation magnetization but reduces the coercivity. This trade-off between saturation magnetization and coercivity is a fundamental challenge in the quest for high-performance exchange-coupled magnets.

**Figure 3:**
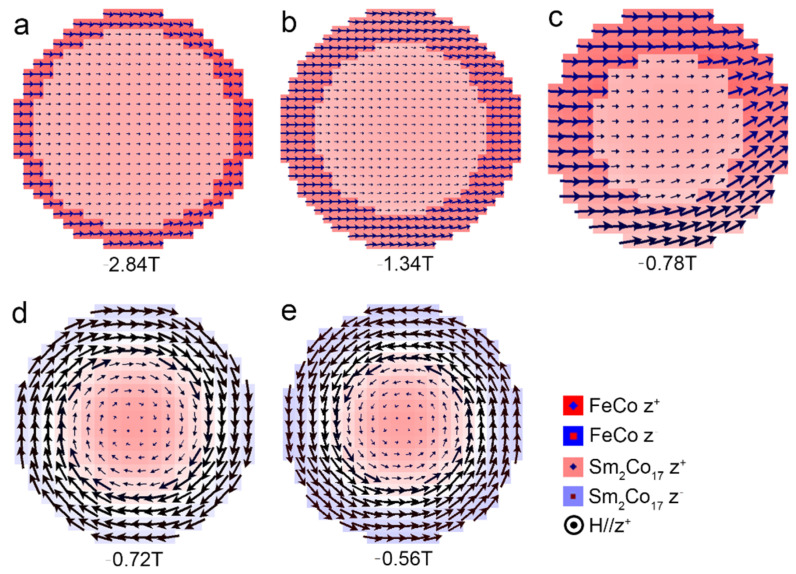
Magnetic structures close to the coercivity of the core/shell cylinders with aspect ratio *L*/*D* = 1 and (**a**) *t* = 1 nm, (**b**) *t* = 2 nm, (**c**) *t* = 3 nm, (**d**) *t* = 4 nm, (**e**) *t* = 5 nm. The observation plane is perpendicular to the applied field (*z*-axis direction).

To gain an in-depth fundamental understanding of the magnetic reversal, we systematically investigated the effect of soft magnetic shell thickness on the magnetic structure evolution during demagnetization. The magnetic structures of different soft magnetic shell thickness prior to coercivity of the core/shell cylinder with *L*/*D* = 1 are shown in Figure 3. As can be seen, the magnetic reversal with different soft magnetic shell thickness exhibits three different characteristics. For the cases of *t* = 1 nm and *t* = 2 nm, the magnetic reversal begins at the soft magnetic shell, and the magnetization reversal mode is coherent rotation. When *t* = 3 nm, magnetization reversal occurs in a quasi-coherent rotation, where the partial magnetic moments of the fringe are tilted. However, when *t* reaches above 4 nm, the magnetization reversal mode evolves into curling [8]. The magnetization reversal mode is determined by the competition between the exchange energy and the demagnetization energy [30]. Generally, the magnetic particles with large size tend towards a reversal in a curling mode, which results in no demagnetization. As the size decreases, the exchange energy density increases, owing to the increase in the relative angle between neighboring magnetic moments. When the size falls into a coherence radius, they tend towards a reversal in coherent-rotation mode. 

### 3.2. Effect of Shape Anisotropy on the Magnetic Properties

**Figure 4:**
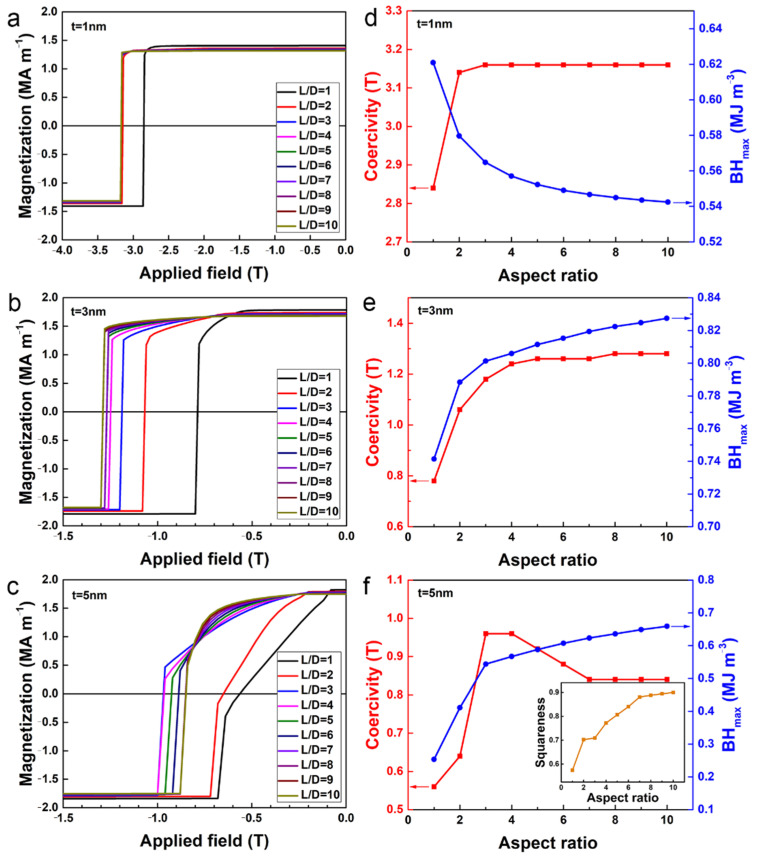
(**a**–**c**) Calculated demagnetization curves of core/shell cylinders for different aspect ratio with (**a**) *t* = 1 nm, (**b**) *t* = 3 nm, (**c**) *t* = 5 nm. (**d**–**f**) The coercivity (red) and maximum magnetic energy product (*BH*)_max_ (blue) of core/shell cylinder with (**d**) *t* = 1 nm, (**e**) *t* = 3 nm, (**f**) *t* = 5 nm, shown as a function of aspect ratio. The inset in (**f**) shows the squareness as a function of the aspect ratio.

Intuitively, the shape anisotropy can be enhanced by elongating the cylinder. To examine the effect of the shape anisotropy of the hard magnetic phase on magnetic properties, we calculated the demagnetization curves of the core/shell cylinders with different aspect ratios. The representative demagnetization curves for the case of *t* = 1 nm, *t* = 3 nm, and *t* = 5 nm are shown in Figure 4a–c, respectively. The coercivity *H*_ci_ and maximum magnetic energy product (*BH*)_max_ deduced from Figure 4a–c are shown as a function of the aspect ratio in Figure 4d–f, respectively. When the thickness of the soft magnetic shell is only 1 nm, as the aspect ratio increases, the coercivity first increases rapidly and then tends to saturate when the aspect ratio reaches above 3 (Figure 4d), and the demagnetization curves with *L*/*D* > 3 are in general overlapped (Figure 4a), suggesting that further enhancing the shape anisotropy would make little contribution to the coercivity. Meanwhile, the increasing aspect ratio of the hard magnetic core leads to a decrease in *V*_soft_ and thus decreases the *M*_s_. As a result, the (*BH*)_max_ decreases monotonously with the aspect ratio increasing (Figure 4d). For *t* = 1 nm, the highest (*BH*)_max_ (0.62 MJ m^−3^, or 78 MGOe) and coercivity (3.16 T) can be achieved with aspect ratio *L*/*D* = 1 and *L*/*D* = 3, respectively. In contrast, when *t* = 3 nm, as the aspect ratio increases, the coercivity first increases and then levels off, and the (*BH*)_max_ increases monotonously with the aspect ratio increasing (Figure 4e). This indicates that shape anisotropy can provide additional coercivity to compensate for the coercivity decreasing caused by soft magnetic shell thickness increasing and thus benefit the optimization of the magnetic energy product. The highest (*BH*)_max_ (0.83 MJ m^−3^, or 104 MGOe) and coercivity (1.28 T) can be both achieved at the aspect ratio of *L*/*D* = 10. For the case of *t* = 5 nm, owing to the too-large volume fraction of the soft magnetic phase, the collapse of magnetization occurred in the core/shell cylinders with a low aspect ratio *L*/*D* ≤ 2 during demagnetization (Figure 4c). As seen from Figure 4f, with the aspect ratio increasing, the (*BH*)_max_ increases monotonously, but the coercivity first increases and then decreases. The partial values of the magnetic energy product and coercivity are as follows: (*BH*)_max_ = 0.54 MJ m^−3^ or 68 MGOe and *H*_ci_ = 0.96 T for *L*/*D* = 3, (*BH*)_max_ = 0.66 MJ m^−3^ or 83 MGOe and *H*_ci_ = 0.84 T for *L*/*D* = 10. The squareness is shown as a function of aspect ratio in the inset of Figure 4f. Here, the squareness is defined as the ratio of the area below the demagnetization curve to the product of remanence *M*_r_ and coercivity *H*_ci_ in the second quadrant, which is also called rectangularity [31]. Clearly, the squareness increases monotonously with the aspect ratio increasing. Based on the Stoner–Wohlfarth model, the enhancement of (*BH*)_max_ may be due to the improvement in squareness of the demagnetization curves [31]. 

**Figure 5:**
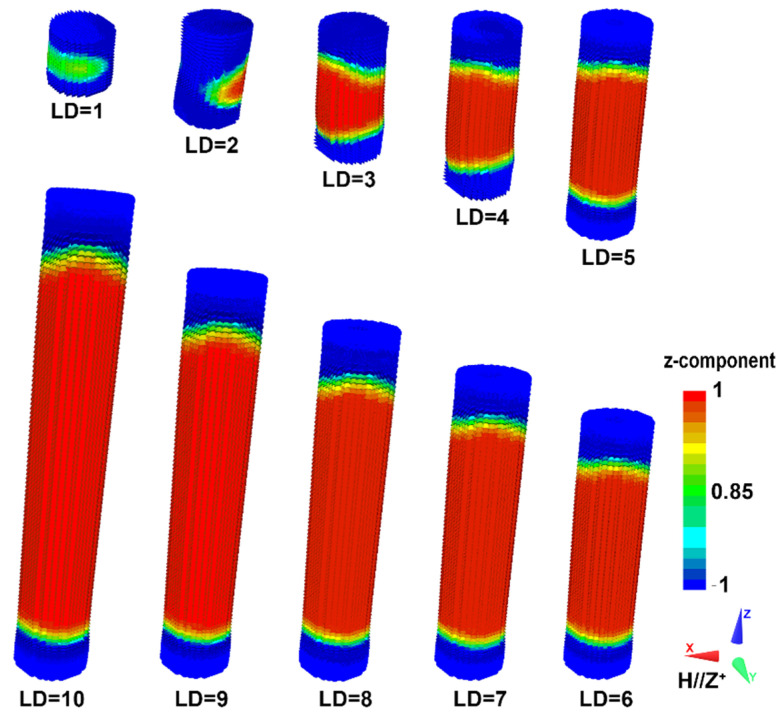
Vector maps of magnetization structure close to the coercivity of core/shell cylinders with different aspect ratios *L*/*D*. The thickness of the soft magnetic shell is 3 nm. The coordinate system and color map indicating the direction of the magnetization are shown next to the image.

In order to understand the details of magnetization reversal mechanisms in core/shell cylinders associated with different aspect ratios, the revolution of the magnetic structures prior to *H*_ci_ was monitored. The representative vector maps of magnetic structures in the core/shell cylinders with *t* = 3 nm are shown in Figure 5. The z-component magnetization in Figure 5 is normalized with respect to its saturation value [32]. Clearly, when the aspect ratio is below the cross-over (*L*/*D* < 3), the magnetocrystalline may be dominant over the shape anisotropy, and the reversal of the magnetic moments along the axis of the cylinder occurs in a quasi-coherent way. However, when the aspect ratio increases to 3, the magnetic reversal starts at the two edges of the cylinder, followed by propagation of two domain walls (head-to-head and tail-to-tail) towards the center [33]. This indicates that at this aspect ratio, the shape anisotropy parallel to the axis of the cylinders dominates, which leads to the increase in coercivity. 

**Figure 6:**
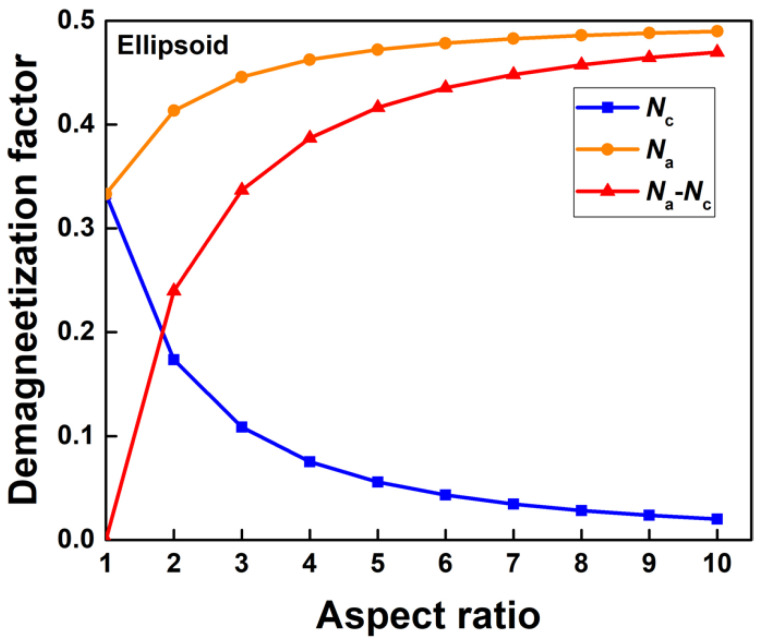
Calculated dependence of the demagnetization factors of an ellipsoid on its aspect ratio.

As mentioned above, the coercivity increases with the aspect ratio, but this effect fades away when the aspect ratio reaches a critical value. The static magnetic behavior of the ellipsoid sets the upper limit of the shape anisotropy coefficient, which helps understand the correlation between the aspect ratio and the shape anisotropy, even the coercivity of the present core/shell cylinders. If no other anisotropy is considered, the magnetic shape anisotropy energy is the total demagnetization energy. In the configuration of the ellipsoid (*c* > *a* = *b*, where *a*, *b*, and *c* are the ellipsoid semi-axes), the coercivity is simply proportioned to the *N*_a_-*N*_c_, where *N*_c_ and *N*_a_ are the demagnetization factors, and satisfying [28]:

*N*_a_ + *N*_b_ + *N*_c_ = 1 (2)

(3) $$N_{c}=\frac{1}{m^{2}-1}\left(\frac{m}{\sqrt{m^{2}-1}}\times \cosh^{-1}(m)-1\right)$$

where *m* = *c*/*a*. The calculated demagnetization factor *N*_a_, *N*_c,_ and *N*_a_-*N*_c_ of the ellipsoid are shown as a function of its aspect ratio in Figure 6. Clearly, the *N*_a_-*N*_c_ first increases rapidly and then increases relatively slowly with the aspect ratio increasing. When the aspect ratio increases approaching 10, further increasing aspect ratio is useless in terms of shape anisotropy optimization [30,33], not to mention the coercivity. 

As demonstrated from the above results, the shape anisotropy contributions to the magnetic properties in the exchange-coupled magnets are closely related to the thickness of the soft magnetic shell. When the soft magnetic shell thickness is very thin, the magnetic properties are dominated by the hard–soft exchange coupling effects, and the contributions of shape anisotropy would be quite limited. Nevertheless, it is also highly challenging to control and manipulate such core/shell exchange coupling magnets, which simultaneously have a large volume fraction of soft magnetic phase and a thin soft magnetic thickness. In the case of a crude core/shell structure with a much thicker soft magnetic shell, the contribution of shape anisotropy emerges and significantly improves the magnetic properties, including the coercivity and magnetic energy product. Considering the flexibility or feasibility of scalable fabrication, utilizing shape anisotropy would be an effective method to improve the magnetic performance of hard–soft exchange-coupled magnets.

### 3.3. The Interaction Effect of Packed Cylindrical Core/Shell Structures

**Figure 7:**
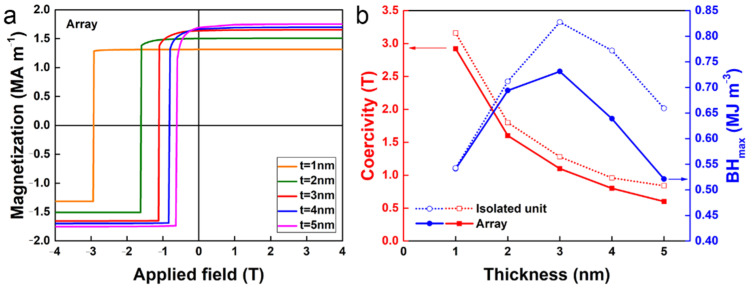
(**a**) Calculated demagnetization curves of the hexagonal close-packed array of core/shell cylinders. (**b**) The intrinsic coercivity and maximum magnetic energy product (*BH*)_max_ shown as a function of the thickness of the soft magnetic shell.

As discussed above, exchange coupling and shape anisotropy has been systematically studied for isolated core/shell cylinders. Nevertheless, the magnetic properties of the entire magnet are determined by those of the unit core/shell cylinder in isolation and the interaction effects due to packing. To examine the effect of packing, we consider a simple hexagonal array of the core/shell cylinders, as illustrated in Figure 1b. Arrays of unit cylinders with geometries of *L*/*D* = 10 are considered, and no periodic boundary conditions were applied in order to avoid artificial interference effects [34]. This simulation is the first extension of a simple hexagonal array of the core/shell cylinders, which will be extended to larger arrays in the future. The demagnetization curves of the hexagonal cylindrical array with different thicknesses of the soft magnetic shell are shown in Figure 7a. The coercivity and maximum magnetic energy product deduced from Figure 7a are shown in Figure 7b. It should be noted that the volume fraction of the soft magnetic phase remains unchanged during packing. Therefore, the assembly of hard core/soft shell cylinders should not experience a reduction in saturation magnetization compared to the isolated unit. However, as seen from Figure 7a, the coercivity of the array is always lower than that in an isolated core/shell cylinder. As a result, the packing effect deteriorates the maximum magnetic energy product, especially when *t* is larger than 2 nm, as shown in Figure 7b. A similar packing effect (or proximity effect) has also been reported for some magnetic nanowires [6]. 

**Figure 8:**
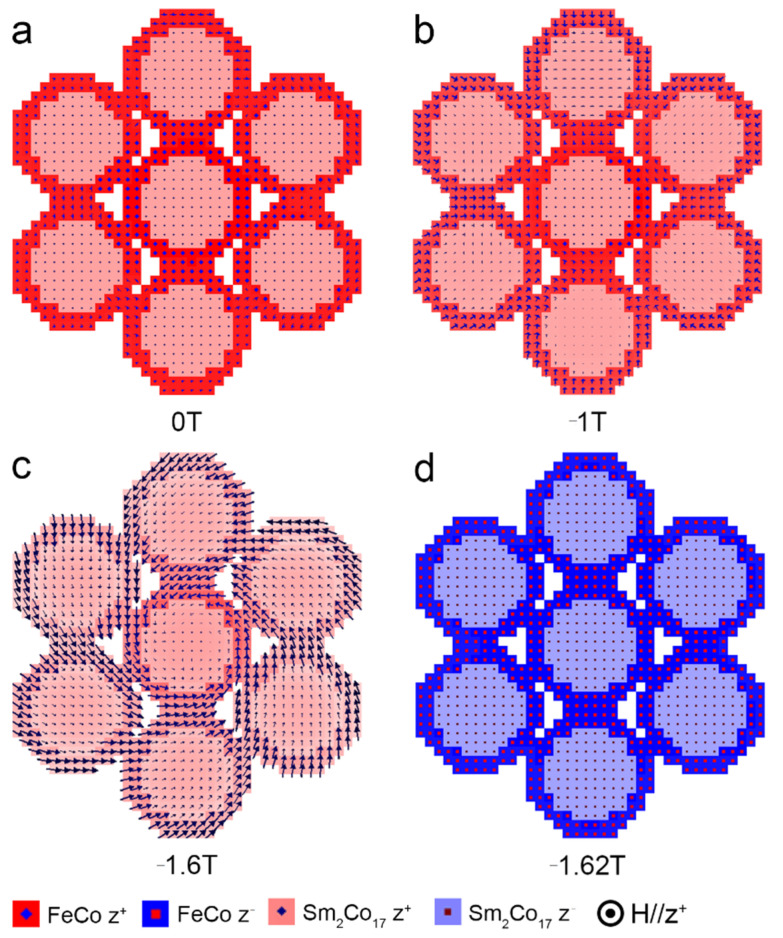
Magnetic structure at the applied field of (**a**) 0 T, (**b**) −1 T, (**c**) −1.6 T, (**d**) −1.62 T during demagnetization for a hexagonal close-packed array of core/shell cylinders with *t* = 2 nm. The observation plane is perpendicular to the applied field.

The representative magnetic structure evolution of the hexagonal close-packed array of core/shell cylinders with *t* = 2 nm during demagnetization is shown in Figure 8. In the hexagonal close-packed array, when *t* ≥ 2 nm, the reversal mechanism of the core/shell cylinder surrounded by an array differs from the magnetic reversal of an isolated cylinder. As seen from Figure 8, the nucleation starts at the soft magnetic shell of the core/shell cylinders in the fringe of the array. Then, a vortex structure is formed, and the center cylinder in the array acts as the core of the vortex. It should be noted that a coherent-rotation mode rather than the curling mode was calculated in the isolated core/shell cylinder with soft magnetic shell thickness of 2 nm. This would be owing to the fact that the single isolated core/shell cylinder only experiences a homogenous external field, while the core/shell cylinder unit in an array is simultaneously affected by the external field and the stray filed of the surrounded cylinders [35], which allows for building a vortex with reduced stray fields in the space between the cylinders [34]. The interaction of each cylinder with the stray field produced by the array would provide an additional antiferromagnetic coupling between the neighboring cylinders, which leads to a decrease in coercivity [36]. Therefore, in order to improve the coercivity of such hard core/soft shell permanent magnets, the units should be separated in a nonmagnetic matric to suppress the proximity effect [12]. 

## 4. Conclusions

In summary, we investigated the geometry, including the thickness of the soft magnetic shell and the aspect ratio of the hard magnetic core, and the packing effect of the cylindrical core/shell hard–soft exchange-coupled magnets by micromagnetic simulations. The underlying magnetization reversal mechanisms were revealed. The following main conclusions can be drawn:

1. As the thickness of soft magnetic shell *t* increases, the saturate magnetization increases monotonously while the coercivity decreases monotonously, and the magnetization reversal changes from coherent rotation mode (*t* ≤ 2 nm) to quasi-coherent rotation mode (*t* = 3 nm), and finally to curling mode (*t* ≥ 4 nm).
2. When the soft magnetic shell is thin (*t* = 1 nm), as the aspect ratio increases, the (*BH*)_max_ decreases monotonously, and there is a trade-off between the *H*_ci_ and (*BH*)_max_. The maximum coercivity of 3.16 T can be achieved at the aspect ratio of 3, and further enhancing the shape anisotropy made little contribution to the coercivity.
3. When the soft magnetic shell is relatively thicker (*t* ≥ 3 nm), as the aspect ratio increases, the (*BH*)_max_ increases monotonously. For *t* = 3 nm, the highest (*BH*)_max_ (0.83 MJ m^−3^, or 104 MGOe) and coercivity (1.28 T) can be both achieved at the aspect ratio *L*/*D* = 10.
4. The packing effect could lead to a decrease in coercivity and (*BH*)_max_ in the hard core/soft shell cylinder array, compared to the isolated one. The magnetization reversal of the cylindrical array occurs in an extraordinary curling mode when *t* ≥ 2 nm, which is different from the isolated one.

This work could provide important guidelines for developing promising hard–soft exchange-coupled permanent magnets with high magnetic properties.

## Data Availability

The data that support the findings of this study are available within the article.
